# Comprehensive transcriptome analysis provides molecular insights into the heterosis-associated drought tolerance and reveals *ZmbHLH137* that promotes drought tolerance in maize seedlings

**DOI:** 10.3389/fpls.2025.1565650

**Published:** 2025-05-23

**Authors:** Liru Cao, Dongling Zhang, Abbas Muhammad Fahim, Huafeng Liu, Zhe Zhang, Desheng Hu, Feiyu Ye, Chenchen Ma, Salah Fatouh Abou-Elwaf, Nora M. Al Aboud, Yinghui Song, Shulei Guo, Qianjin Zhang, Xin Zhang, Xiaomin Lu

**Affiliations:** ^1^ The Shennong Laboratory, Grain Crops Research Institute, Henan Academy of Agricultural Sciences, Zhengzhou, Henan, China; ^2^ College of Agronomy, Gansu Agricultural University, Lanzhou, Gansu, China; ^3^ Zhengzhou No.9 High School International Department, Zhengzhou, Henan, China; ^4^ Agronomy Department, Faculty of Agriculture, Assiut University, Assiut, Egypt; ^5^ Department of Biology, Faculty of Science, Umm Al-Qura University, Makkah, Saudi Arabia

**Keywords:** *Zea mays*, heterosis, ROS scavenging, RT-qPCR, WGCNA, RNA-seq, bHLH

## Abstract

Drought, a primary environmental factor, imposes significant constraints on maize’s developmental processes and productivity. Heterosis breeding is one of the most important breeding strategies for reducing drought-induced yield losses. The genetic mechanisms of heterosis for drought tolerance in maize remain unclear to date. This study aims to analyze the expression profiles and potential heterosis-related genes of the ZhengDan618 hybrid (F_1_) and its parents, Zheng8713 (parental parent) and ZhengC126 (maternal parent), with extreme differences in drought tolerance under well-irrigated (WI) and drought-stressed (DS) conditions by RNA-sequencing. F_1_ plants exhibited the strongest antioxidant enzyme activity and drought tolerance, followed by the parental parent. Transcriptome analysis revealed 1,259 unique differentially expressed genes (DEGs) in the F_1_ hybrid after drought stress induction, mainly involved in the “Glutathione metabolism” and “Flavonoid biosynthesis” pathways. There were fewer DEGs between the F_1_ and the parental parent, with the drought tolerance phenotype mostly attributed to the contribution of the drought-tolerant parent Zheng87. The weighted gene co-expression network analysis combined with non-additive gene mining identified 13 non-additive drought stress-associated genes, among them *bHLH137* expression exhibited up-regulated expression in response to drought stress. Under drought stress, *ZmbHLH137*-overexpressing maize plants revealed the lowest H_2_O_2_ and MDA content, followed by the B104 WT plants, whereas the *zmbhlh137* knockout mutants exhibited the highest H_2_O_2_ and MDA content. Moreover, *ZmbHLH137*-overexpressing maize plants exhibited the higher glutathione peroxidase, catalase, peroxidase, and superoxide dismutase activities, whereas the *zmbhlh137* knockout mutants exhibited the lower oxidase activity. These results indicate that *ZmbHLH137* positively regulates drought tolerance in maize at the seedling stage by regulating antioxidant enzyme activity. These findings provide novel insights into heterosis regulation in maize seedlings. The identified genes are important genetic resources and may aid strategies for improving drought tolerance in maize.

## 1 Introduction

Maize (*Zea mays* L.), the 3^rd^ most important crop worldwide, is essential for food and feed. In light of global climate change, maize production is threatened by various biotic and abiotic stresses (Klopfenstein et al., 2013; Badr et al., 2020). In most maize-growing areas, drought stress or water deficit is one of the major environmental factors causing substantial yield reduction (Tollenaar and Lee, 2002; Wang et al., 2016; Cao et al., 2021). The use of crop plants’ biological and genetic capabilities to increase agricultural productivity under the same water supply conditions is termed biological water saving. The primary objective of biological water saving is to achieve genetic improvement by breeding new varieties that are resistant to drought or require less water (Cao et al., 2021). Heterosis refers to the phenomenon where hybrids exhibit superior phenotypes compared to their parents in agronomic characteristics environmental adaptability and stress resistance. Maize is a key species for heterosis research and demonstrates considerable heterosis in biomass, plant height, root growth, photosynthesis, starch metabolism, grain yield as well as biotic and abiotic stress resistance (Lee and Tollenaar, 2007; Schnable and Springer, 2013). Heterosis, as a breeding technique, has been extensively utilized to enhance the productivity and quality of numerous crop species (Kempe et al., 2014; Fujimoto et al., 2018). The commercial exploitation of this phenomenon began in maize with the development of the first high-yielding hybrid cultivar, known as the double-cross hybrid maize (Funk 250), in 1922 (Troyer, 2009). Since 2011, the maize yield in America has increased by a minimum of eightfold, primarily as a result of cultivating hybrid varieties (Feng et al., 2011).

The precise mechanism of heterosis has remained perplexing, which has impeded the proper utilization of heterosis. Non-additive genes in hybrids have received significant attention and are recognized as the primary genes responsible for heterosis in the progeny (Luo et al., 2001; Liu et al., 2020). Non-additive genes can be classified into two categories, i.e., hybrid genes that are significantly higher or lower than both parents, referred to as overexpressed genes, and hybrid genes that are similar to the highly expressed parent but significantly higher than or similar to the low-expressed parent but significantly lower than the highly expressed parent, known as dominant genes. Moreover, dominance, over-dominance, and epistasis have been suggested as possible genetic explanations for the heterotic characteristics observed in the F_1_ generation (Davenport, 1908; Jones, 1917; Stuber et al., 1992; Yu et al., 1997). However, traditional genetic approaches have only revealed a limited number of heterosis loci functioning in these modes (Liu et al., 2024). Recently, the advancement of efficient molecular detection and quantification techniques has provided molecular-level evidence in favor of these genetic ideas for various traits across multiple species (Liu et al., 2021).

Over the past two decades, high throughput sequencing and bioinformatics tools have rapidly characterized heterosis genetic and molecular bases. Several innovative strategies have proven effective in analyzing the mechanism of heterosis (Schnable and Springer, 2013; Fujimoto et al., 2018). Genome, epigenome, transcriptome, proteome, and metabolome profiles of hybrid combinations in diverse species have shown significant biological pathways or genes related to heterosis (Petrizzelli et al., 2019; Shao et al., 2019). These findings suggest that the underlying principles of heterosis vary depending on the specific combinations and characteristics being examined. However, certain studies have provided valuable insights into the molecular mechanisms that control gene expression associated with heterosis in hybrids (Liu et al., 2024). A study on the genetics of flag leaf heterosis in the Chinese indica super hybrid rice WFYT025 revealed that four *WRKY* transcription factors (TFs) have the potential to influence both grain quantity and photosynthesis (Cheng et al., 2023). The transcriptome data of foxtail millet (*Setaria Italica* L.) hybrid Zhangza-19 and its parents before and after drought stress showed that *SiMYBS3*, a drought-related heterophytic gene, positively regulates millet drought resistance (Liu et al., 2023). In 2009, the reference genome sequencing of maize B73 represented a major step in molecular maize research (Schnable et al., 2009). Transcriptome sequencing has been extensively employed in the investigation of maize heterosis. It has been reported that the senescence of ear leaves was significantly delayed after silk spinning by the hybrid combination of B73/Mo17 and Zheng 58/Chang 7-2, the study was conducted to investigate the regulatory impact of heterosis on the senescence of maize ear leaves. The findings revealed that genes associated with photosynthesis and starch biosynthesis (*ZmAPS1*, *ZmAPL*) exhibited dominant expression, suggesting their involvement in regulating senescence and heterosis regulatory networks (Song et al., 2016). Recent investigations have shown that the ZhengDan958 hybrid exhibits heterosis during germination and develops seed imbibition faster than its parent. Transcriptional analysis has found that genes highly expressed in metabolic pathways such as carbon metabolism, glycolysis/gluconogenesis, and endoplasmic reticulum protein processing are major contributors to the heterosis of maize seed germination (Wan et al., 2022).

Previous studies on maize heterosis have focused on phenotypic and molecular pathways related to growth and development (Chen, 2013; Song et al., 2016). The precise molecular mechanism behind the heterosis in maize under abiotic stress conditions remains unknown. In this study, a comprehensive analysis was performed to assess the drought resistance of the ZhengDan618 hybrid (F_1_), paternal parent Zheng8713 (F), and maternal parent ZhengC126 (M), by integrating phenotypic, physiological, biochemical, and transcriptomics analysis under both well-irrigated (WI) and drought-stressed (DS) conditions. This study aims to: i) determine whether heterosis arises from paternal or maternal origin, and ii) identify the key genes that contribute to heterosis and are associated with drought tolerance by WGCNA, GO, and KEGG enrichment analysis. Our findings elucidated the primary pathways involved in heterosis-associated drought tolerance. Furthermore, the candidate genes responsible for the heterosis-associated drought tolerance in maize were determined and verified by RT-qPCR. Subsequent functional analysis of candidate genes was performed to verify the function of these genes. The mechanism by which candidate genes regulate the hybridization of maize drought tolerance was further investigated by analyzing phenotypic, physiological, and biochemical changes resulting from well-irrigation and drought stress treatments. The findings of this study offer genetic resources for enhancing the molecular characteristics of maize to tolerate drought and also serve as a great source for comprehending the heterosis-associated drought tolerance in maize.

## 2 Materials and methods

### 2.1 Plant materials, growth conditions, and drought treatment

The highly drought-tolerant paternal parent, Zheng8713 (F), and the drought-sensitive maternal parent ZhengC126 (M), along with their F_1_ cross hybrid ZhengDan618 were used in this study. Moreover, the B104 maize inbred (WT), *ZmbHLH137* overexpression transgenic (*ZmbHLH137*-OE) plants, and the *ZmbHLH137* knockout mutant in the B104 background were used. Maize genetic transformation was carried out by Beijing Bomeixingao Technology Company. Seeds of all genotypes were grown in a greenhouse at 28°C under long days (LD) conditions at 16/8h light/dark cycle. Seeds from Zheng8713, ZhengC126, and their F1, the B104 maize lines overexpressing 35S::*ZmbHLH137* (*ZmbHLH137*-OE), *zmbhlh137* knockout mutant, and the B104 (WT) were sown in 21 × 16 × 13 cm pots filled with the nutrient soil and vermiculite mixture (3:1) and grown at 25-28°C, relative humidity of 80% and a 16/8 h light/dark cycle. Healthy and uniform plants were selected and subjected to drought stress and well-irrigation. The drought stress(DS) treatment involved cultivating three‐leaf old maize seedlings in soil under standard greenhouse conditions, followed by a 7‐day period of with held watering to induce drought stress. At the same time, we applied well-irrigation(WI) to the three-leaf old maize seedlings and watered it 200 ml per day until the DS ended, After 7 days of DS, maize leaves from transgenic and WT plants were collected and immediately frozen in liquid nitrogen, and subsequently stored at -80°C for all the above-ground leaves, it was used for RNA-Seq, qRT-PCR, determination of chlorophyll content and relative water content of leaves. Three biological replicates were performed for each treatment.

### 2.2 RNA isolation, library preparation, RNA-seq, WGCNA and RT-qPCR

Total RNA was isolated from maize leaves using the RNeasy Plant Mini Kit (Qiagen), and purified using the magnetic stand (Invitrogen). The integrity and purity of RNA libraries were assessed using the 2100 Bioanalyser (Agilent Technologies in Santa Clara, CA, USA), and the ND-2000 from NanoDrop Technologies, respectively. The Illumina standard protocol (TruSeq Stranded RNA LT Guide) was employed to prepare cDNA libraries. The RNA transcriptome libraries of high quality were compared using the TruSeqTM RNA kits from Illumina Inc. (San Diego, CA, USA). The Illumina HiSeq4000 system was performed to conduct RNA sequencing (RNA-seq) according to the manufacturer’s instructions. Further RNA-Seq analysis was performed as described in our previous study (Cao et al., 2021). The Fastp (https://github.com/OpenGene/fastp) online tool was employed to process raw reads to produce clean data. The HISAT2 software (v2.1.0) was used to align the obtained clean reads to the B73 maize genome version 4. The cufflinks online tool (http://cole-trapnell-lab.github.io/cufflinks/releases/v2.2.1/) was used to count gene expression and report it in fragments per kilobase of transcript per million fragments mapped (FPKM). The FPKM value is a measure of the transcript expression normalized to the transcript length and the total number of fragments. The glmTreat function in the R package (edgeR v3.40.2)was employed to perform significance analyses. The glmTreat function assesses whether the changes in the differential expression fold are significantly greater than 1.5. Significant differentially expressed genes (DEGs) with more than two-fold change and a false discovery rate (FDR) of ≥0.05 were selected for further analyses.

The agriGO v2.0 (http://systemsbiology.cau.edu.cn/agriGOv2/index.php) online tool was used to perform GO enrichment analysis of the identified DEGs. The gene ontology type was set as Plant GO slim in the agriGO v2.0. The Kyoto Encyclopedia of Genes and Genomes (KEGG) database was then employed to annotate the pathways of DEGs. The online bioinformatics tools KAAS of the KEGG (https://www.genome.jp/kegg/kaas/) were employed for the annotation of gene descriptions in the KEGG database. The KEGG mapper online service tool (https://www.genome.jp/kegg/mapper.html) was used to map the annotation results to the KEGG pathway database. Fisher’s exact test was utilized to determine significance levels. The B‐Y method (Benjamini & Yekutieli, 2001) was used for multiple testing corrections by the agriGO v2.0. Results with adjusted p-values ≤ 0.05 were identified as significantly enriched GO terms. Pathway enrichment analysis was performed using the OmicShare (www.omicshare.com/tools) online tool to identify significantly enriched metabolic pathways or signal transduction pathways in differentially expressed genes. Significantly enriched pathways in differentially expressed genes were defined by hypergeometric test, by taking FDR<0.05 as a threshold. The weighted gene co-expression network analysis (WGCNA) analysis of DEGs identified by RNA-Seq was performed using the R package (Version 3.4.4) to construct a gene co-expression network. The Cytoscape software (Version 3.10.1) was implemented to depict the network. The pickSoftThreshold tool in the R Package was used to calculate soft power and build the co-expression network with β = 10. A mergeCutHeight value of 0.25 was selected to merge modules that exhibited more than 75% similarity. Subsequently, genes with comparable expression patterns were merged into the same module.

In the present study, we selected a kME of more than 0.7 between gene expression and module eigenvalues for further study. These genes are more effective in representing the overall expression pattern of the entire module. Furthermore, to validate differential expression between the *ZmbHLH137*-OE lines and the WT plants identified by RNA-Seq, 20 differentially expressed genes (DEGs) were randomly selected for expression analysis using the quantitative real‐time PCR (RT-qPCR). The RT-qPCR analysis was performed using a Light Cycler 480 instrument (Roche, Basel, Switzerland) using Hieff^®^qPCR SYBR^®^Green Master Mix (YEASEN, Shanghai, China) according to the manufacturer’s instructions. Relative gene expression was determined using the 2^-ΔΔCt^ approach (Pfaffl, 2001) and using ANOVA for gene differential significance analysis. Expression levels were assessed in three technical and biological replicates and normalized against the 18S ribosomal gene. The primer sequences used in the RT-qPCR assay are shown in **Supplementary Table S1**.

### 2.3 Construction of transgenic lines for functional analysis

For the development of 35::*ZmbHLH137*-overexpressing (*ZmbHLH137*-OE) maize plants, the complete coding sequence (CDS) of *ZmbHLH137* was amplified from the B73 cDNA using sequence-specific primers (**Supplementary Table S1**). The TaKaRa hi-fi enzyme (TAKARA, Dalian, China) was used for the amplification of *ZmbHLH137*. Amplicon was then ligated into the pMD-19T vector (TAKARA, Dalian, China), and then transformed into *Escherichia coli* DH5α strain. The CDS of *ZmbHLH137* was then inserted into the pFGC5941 binary vector under the control of the cauliflower mosaic virus (CaMV) 35 S promoter (35 S::*ZmbHLH137*). Enzymatic restriction digestion, PCR, and sequencing then verified the intactness of the binary vector to the CDS of *ZmbHLH137*. The recombinant vector (pFGC5941-35S::*ZmbHLH137*) was then transformed into the GV3101 *Agrobacterium tumefacien* competent cells for genetic transformation. The Bo Mei Xing Ao Technology Co., Beijing was employed for maize genetic transformation using the pFGC5941-35S::*ZmbHLH137* recombinant vector in the maize B104 inbred line.

*Zmbhlh137* maize knockout mutants were generated using a CRISPR/Cas9-mediated mutagenesis technology. The online tool CRISPR-PLANT (http://www.genome.arizona.edu/crispr/CRISPRsearch.html) was employed to select the gRNAs (targeting sequences) (**Supplementary Table S1**). Vector construction was performed as described by (Xing et al., 2014). The PCR fragment was amplified from pCBC-MT_1_T_2_ using the ZmbHLH137-F1/F2 and ZmbHLH137-R1/R2 primers. The gRNA was inserted into the pBUE411 vector between the *BsaI* sites of the pBUE411 vector by Golden Gate cloning, resulting in pBUE411-*ZmbHLH137*. The pBUE411-*ZmbHLH137* recombinant vector was introduced into immature zygotic embryos of the B104 maize inbred line harvested 9 days after pollination by *A. tumefaciens*-mediated transformation following the procedure described by (Frame et al., 2002). The knockout mutant lines were obtained from T_0_ lines, and plants without any sequence editing were selected as the WT lines and used for subsequent studies.

### 2.4 Phenotypic evaluation, chlorophyll content, and antioxidant enzyme activity

Phenotypic evaluation of Zheng8713, ZhengC126, and their F1, *ZmbHLH137*-OE, *zmbhlh137* knockout mutant, and the wild-type plants was performed under normal and drought-stressed conditions for 7 days. A portable chlorophyll meter (YD-LA, Laiende, China) was employed to measure the chlorophyll content of maize leaves. The relative water content of leaves (RWC) was calculated by determining the fresh weight (FW), dry weight (DW), and saturated weight (SW) according to the following formula:


$$RWC=\frac{\left(FW-DW\right)}{\left(SW-DW\right)}\times 100$$

The rate of water loss for each plant was calculated over 5 hours.

A reaction mixture consists of 0.3 ml nitroblue tetrazolium (NBT, 750 µM), 1.5 ml PBS (0.05 M, pH 7.8), 0.3 ml methionine (130 mM), 0.3 ml EDTA-Na2 (100 µM), 0.3 ml riboflavin (VB2, 20µM), and 0.25 ml ddH_2_O was implemented to measure the activity of superoxide dismutase (SOD) by detecting the absorbance at 560 nm. For measuring the content of Malondialdehyde (MDA), 0.6% thiobarbituric acid solution dissolved in 10% trichloroacetic acid (TCA). An aliquot of 1 ml of the initial enzyme solution was added to 2 ml of the mixture. MDA content was then detected at 600, 532, and 450 nm using a spectrophotometer. The concentration of hydrogen peroxide (H_2_O_2_) was quantified using the ferrous oxidation-xylenol orange (FOX) assay. The activity of peroxidase (POD) was assessed in a reaction mixture of 2.5 ml PBS (0.1 M, pH 6.0), 2.8 µl guaiacol, and 19 µl H_2_O_2_ (30%). The absorbance was detected at 470 nm and a kinetic scan for 3 min. The extraction of catalase (CAT) was carried out using a 0.05 M phosphate buffer solution that contained 1% polyvinylpyrrolidone (PVP) at a pH of 7.5, and then the enzyme activity was determined. The activities of glutathione peroxidases (GPX) (Kit No. BC1190) were detected according to the manufacturer’s instructions (Solarbio, China).

## 3 Results

### 3.1 Variations of parental line and their cross hybrid under drought-stressed conditions

**Figure 1:**
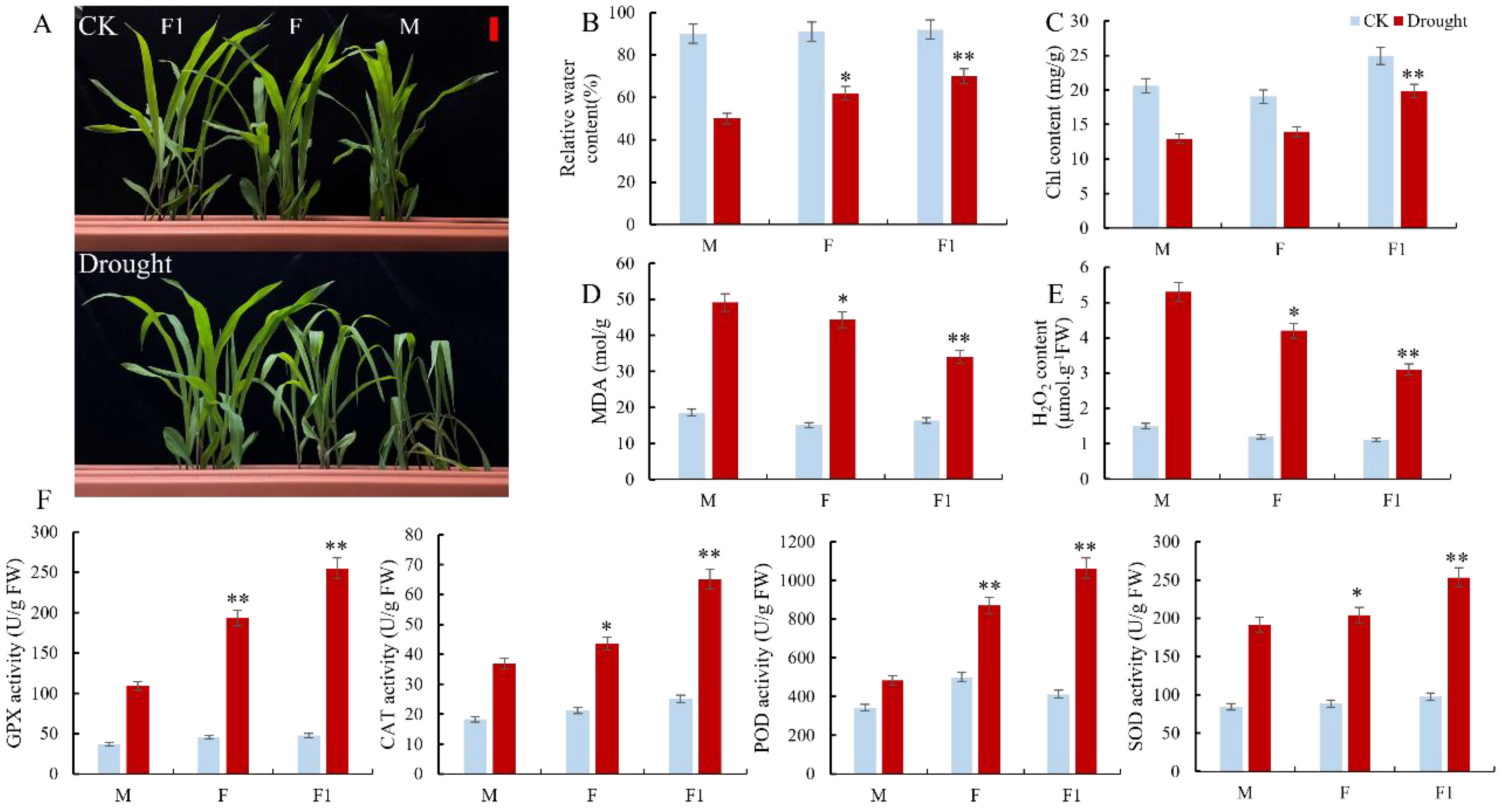
The Phenotypic characterization of hybrid, paternal and maternal lines under well-irrigated (WI) and drought-stressed (DS) conditions. **(A)** The phenotypes of hybrids (F_1_; left), paternal (F; middle), and maternal (M; right) lines under normal and drought stress. The scale bars with red color are 5.0 cm. **(B-E)** Relative water content, chlorophyll content, MDA content, and H_2_O_2_ content in the leaf of the F_1_, F, and M plants (n = 3, ± SD). **(F)** Changes of GPX, CAT, POD, and SOD activities in F_1_, F, and M plants (n = 3, ± SD).One-way ANOVA was used to compare F1, F, and M under DS and WI: * and ** represent differences at 0.05 and 0.01 significance levels between F_1_, F and M, respectively.

The paternal parents Zheng8713 and ZhengC126 and their F_1_ cross hybrid ZhengDan618 exhibited normal growth and development under well-irrigated conditions. However, after 7 days of drought stress, the growth of both parents was significantly affected, particularly the drought-sensitive maternal parent ZhengC126, which displayed leaf wilting and minor chlorosis compared to the F_1_ hybrid (**Figure 1A**). These results illustrate that the paternal parent Zheng8713 may contribute significantly to improving drought tolerance in the F_1_ hybrid. Moreover, we measure the physiological and biochemical changes in both parental lines and their cross-hybrid under well-irrigated (WI) and drought-stressed (DS) conditions. Compared to the WI conditions, the relative water content (RWC) of Zheng8713, ZhengC126, and ZhengDan618 was significantly decreased by 22, 29, and 40%, respectively, under the DS conditions (**Figure 1B**). Similarly, the leaf chlorophyll content exhibited a similar pattern as the RWC in the parental lines and the F_1_ hybrid. However, the F_1_ hybrid showed the least decline, followed by Zheng8713, while the drought-sensitive maternal parent ZhengC126 exhibited the most significant reduction (**Figure 1C**). The accumulation of reactive oxygen species (ROS) caused by drought can harm cell membranes. Malondialdehyde (MDA) serves as an important indicator of plant cell membrane peroxidation. The results indicate that, under DS conditions, the MDA content in the Zheng8713, ZhengC126, and ZhengDan618 was increased by 18, 29, and 30%, respectively, compared to the WI conditions (**Figure 1D**). The results suggest that drought caused the most damage to the cell membrane of the drought-sensitive maternal parent ZhengC126 followed by the drought-tolerant parent Zheng8713, and had the least effect on the F_1_ hybrid. Furthermore, H_2_O_2_ content in both the parental and hybrid lines exhibited a similar variation trend to that observed for MDA (**Figure 1E**). Antioxidant enzymes play a significant role in removing reactive oxygen species (ROS) and are essential for plants to withstand stress. The activities of glutathione peroxidase (GPX), catalase (CAT), peroxidase (POD), and superoxide dismutase (SOD) were significantly increased in the F_1_ hybrid by 432, 159, 158, and 159%, respectively, in the drought-tolerant parent Zheng8713 by 323, 105, 73, and 129%, and the drought-sensitive parent ZhengC126 196, 102, 41, and 127%, respectively (**Figure 1F**). The results illustrate that the F_1_ hybrid mitigates cell damage and enhances drought tolerance by enhancing the activity of antioxidant enzymes during drought stress. Compared to its parents, the F_1_ hybrid exhibited a greater drought tolerance attitude, highlighting the impact of heterosis. Furthermore, the drought-tolerant parent Zheng8713 exhibits greater tolerance to drought stress compared to the drought-sensitive parent ZhengC126, suggesting that the heterosis impact might be attributed to the drought-tolerant parent Zheng8713.

### 3.2 Transcriptome and RT-qPCR analyses of drought tolerance in the F_1_ hybrid

Based on phenotypic and physiological analyses, there was a significant increase in drought tolerance in the F_1_ hybrid, with the drought tolerance phenotype mostly attributed to the contribution of the drought-tolerant parent Zheng871. To study the differences in the response mechanisms to drought stress between the F_1_ hybrid and its parental lines, total RNA was extracted from leaves to construct 18 libraries. As a result, 823.89 million raw reads were generated with an average of 45.77 million per sample. After the removal of low-quality reads, the total number of effective clean reads was 819.03 million, and the average number of clean reads per sample was 45.5 million. In each sample, Q20 exceeded 95.75% and the percentage of Q30 bases exceeded 89.38% (**Supplementary Table S2**). The analysis showed that the samples were well-preserved, and the data quality was high and met the experiment requirements. Principal component analysis (PCA) of the samples from the F_1_ and its parents before and after drought stress treatment showed that the similarity between treatments was high, and the biological repetitive clustering effect was significant in the control and treatment groups. Principal component 1 (PC1) explains 80% of the transcriptome variations, and principal component 2 (PC2) explains 11.2% of the principal component variations (**Supplementary Figure S1A**). Correlation heat maps showed strong correlation and repeatability between samples within the same group (**Supplementary Figure S1B**). Gene expression was quantified using FPKM. An analysis of the FPKM distribution of the 18 samples using a box plot indicates that the sequencing results were relatively reliable and that the same material exhibits excellent consistency in terms of overall sample expression (**Supplementary Figure S1C**).

**Figure 2:**
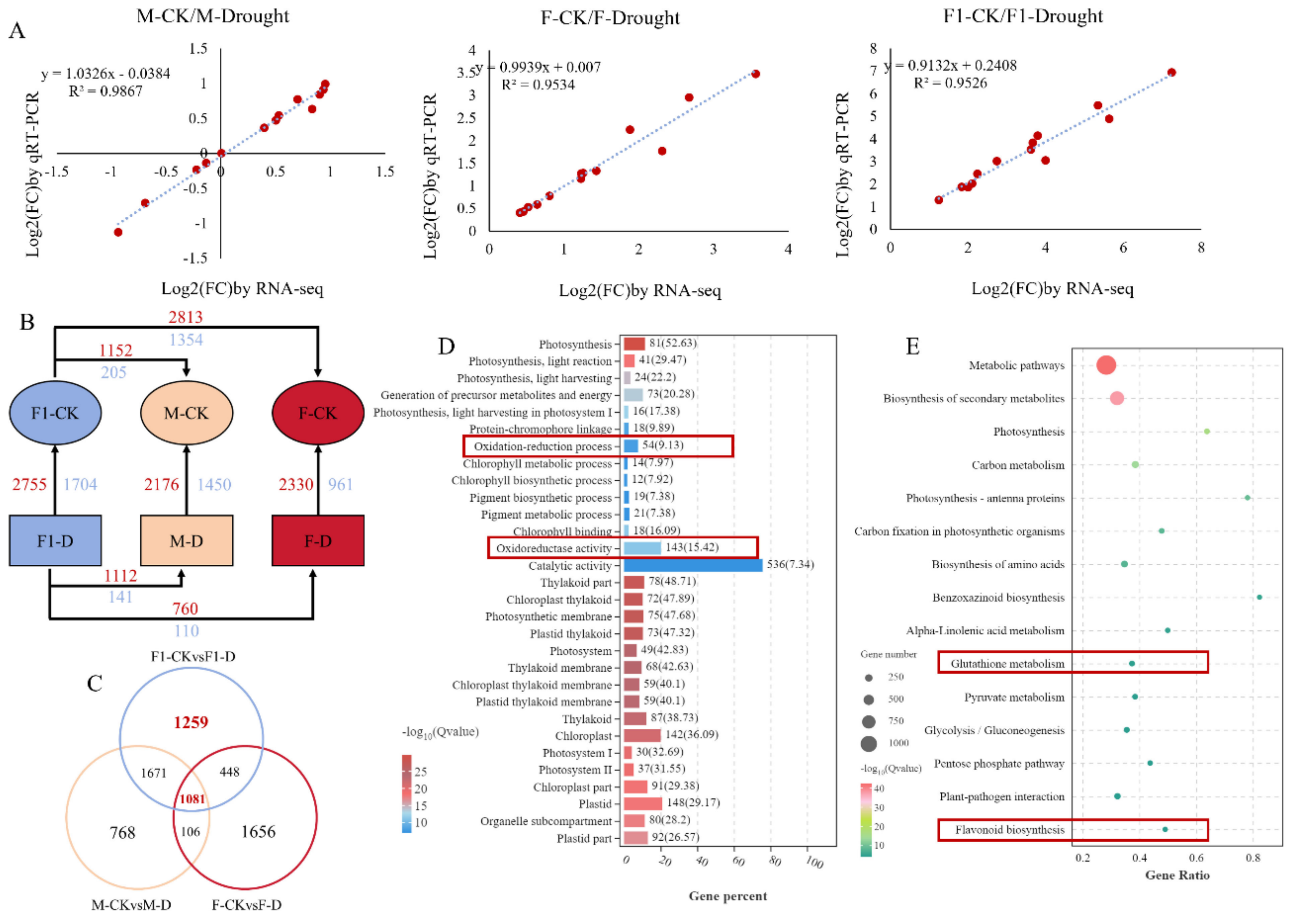
Transcriptomic analysis of the F_1_, parental (F), and maternal (M) lines well-irrigated (WI) and drought-stressed (DS). **(A)** Validation of expression differences observed by RNA-seq through quantitative PCR of 13 differentially expressed genes (DEGs). **(B)** Numbers of DEGs in different paired comparisons. Red and blue numbers indicate up- and downregulated genes, respectively. **(C)** Venn diagrams of overlapping and non-overlapping genes among the different comparisons. **(D)** Gene Ontology enrichment analysis of common DEGs of F_1_, F and M strains under WI and DS conditions. **(E)** Under WI and DS conditions, the KEGG (Kyoto Encyclopedia of Genes and Genomes) pathway corresponding to special differentially expressed genes (DEGs) that were significantly enriched in F_1_ plants was analyzed. Specific comparisons include: ‘F_1_-WI vs. F_1_-DS’, that is, the comparison between the F_1_ well-irrigated control group and the F_1_ drought stress group; “F-wi vs. F-DD”, that is, the comparison of F good irrigation control group and F drought stress group; And ‘M-WI vs. M-DS’, that is, the comparison between the M well-irrigated control group and the M drought stress group.

To further confirm the accuracy and repeatability of the RNA-Seq results, 13 genes were randomly selected for RT-qPCR analysis. The observed trend in the RT-qPCR results was in agreement with the pattern observed in the RNA-seq data (**Figure 2A**), indicating the reliability of the results of transcriptome sequencing. Furthermore, the genes with a log2-fold change greater than 2 were classified as differentially expressed genes (DEGs). We observed that the F_1_ hybrid exhibits a total of 4,459 DEGs either before and after drought stress induction, from which 2,755 genes showed up-regulated expression and 1,704 genes showed down-regulated expression. There were 3,291 DEGs identified in the drought-tolerant parent Zheng871, of which 2,330 were upregulated and 961 were downregulated. A total of 3,626 DEGs were detected in the drought-sensitive parent ZhengC126, including 2,176 upregulated and 1,450 downregulated genes (**Figure 2B**). Venn diagram showed that 1,081 DEGs were shared between the F_1_ hybrid and its parental lines (**Figure 2C**). The GO analysis of the 1,081 genes revealed their primary involvement in photosynthesis, chlorophyll metabolism, and oxidoreductase processes, in particular, the oxidation-reduction process (GO: 0055114) and “ oxidoreductase activity (GO: 0016491) were related to antioxidant processes. This pathway may play a crucial role in the response of maize to drought stress (**Figure 2D**). An analysis was conducted to identify the differential genes between the F_1_ hybrid and its parents. Under WI conditions, there were 1,357 DEGs between the F_1_ hybrid and the drought-tolerant parent Zheng8713 (1,152 upregulated and 205 downregulated), and 4,167 DEGs between the F_1_ hybrid and the drought-sensitive parent ZhengC126 (2,813 upregulated and 1,354 downregulated) (**Figure 2B**). Following DS conditions, a total of 870 DEGs were observed between the F_1_ hybrid and the drought-sensitive parent ZhengC126, with 760 genes upregulated and 110 genes downregulated. Similarly, 1,253 DEGs between the F_1_ hybrid and the drought-tolerant parent Zheng8713 with 1,112 genes upregulated and 141 genes downregulated. Notably, the smallest number of DEGs was found between the F_1_ hybrid and ZhengC126 (**Figure 2B**), suggesting that the drought-tolerant paternal parent Zheng8713 contributes significantly to the enhanced drought tolerance phenotype of the F_1_ hybrid.

Additionally, the KEGG pathway analysis provides a classification of the complex biological functions of the genes of interest. As shown in Venn diagram, 1,259 DEGs were found unique to the F_1_ hybrid (**Figure 2C**), which were enriched in pathways such as “ Glutathione metabolism “ and “ Flavonoid biosynthesis “ (**Figure 2E**), both were found to play an important role in protecting plants from oxidative damage. Besides, the results revealed that GPX enzyme activity in the F_1_ hybrid is significantly higher than that in the parents in response to drought stress. The results demonstrates the implication of antioxidant mechanisms in regulating the response of the F_1_ hybrid to drought stress and heterosis.

### 3.3 Comparative analysis of non-additive genes in the F_1_ hybrid

**Figure 3:**
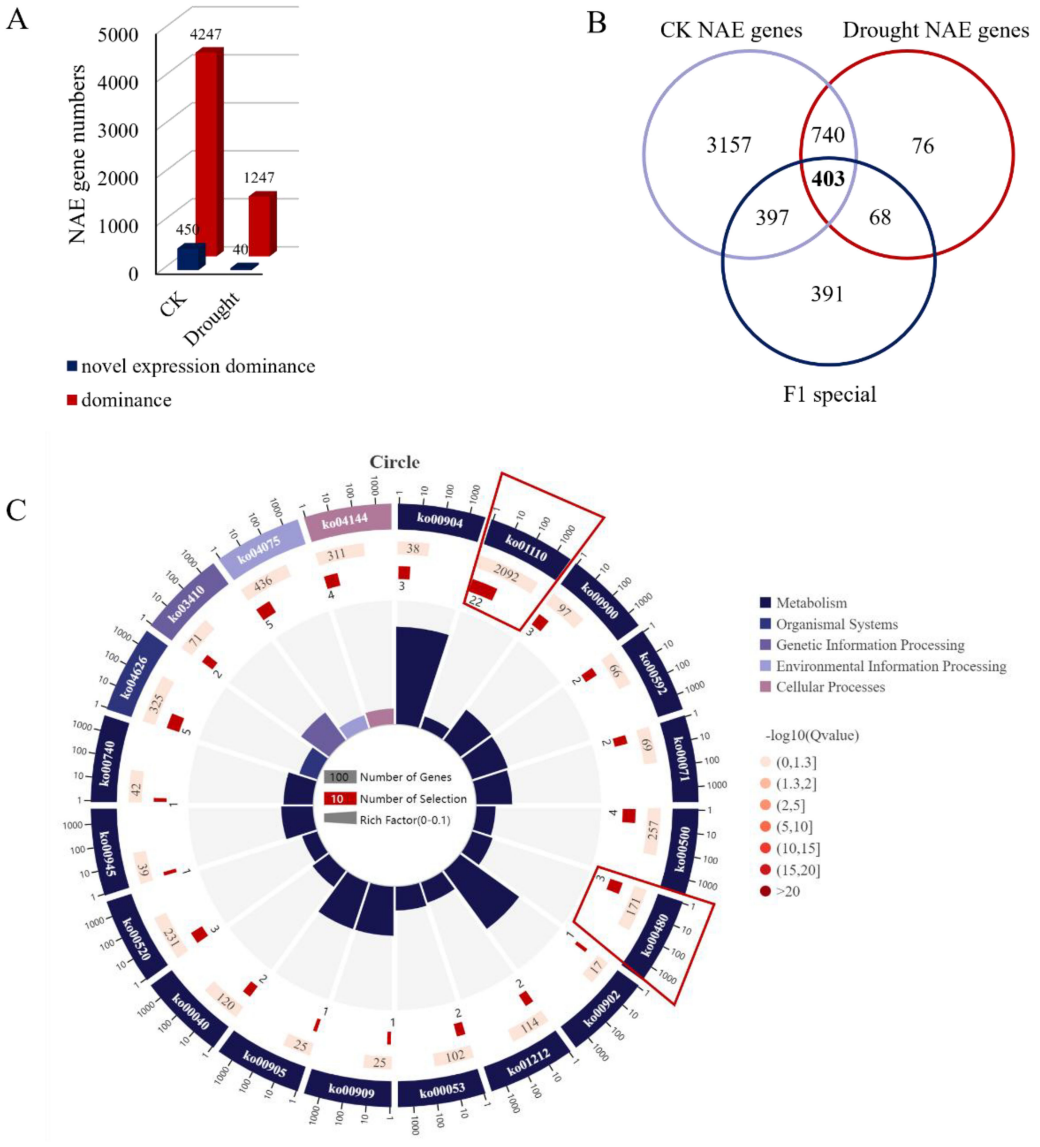
Identification of genes showing nonadditive expression (NAE) in the F_1_ hybrid line. **(A)** Number of genes showing NAE in the F_1_ under normal and drought conditions. Red and blue represent over-dominant and dominant patterns, respectively. **(B)** Venn diagram showing the overlap between NAE genes in normal and drought stress conditions specific to the F_1_ in response to drought stress. **(C)** KEGG pathway enrichment analysis among the 403 genes showing NAE specifically in the F_1_ in response to drought stress.

To elucidate how changes in gene expression affect drought-resistant heterosis, we studied genes with significantly different expression patterns between the F_1_ hybrid and its parents and found non-additive expression (NAE) of hetero-associated genes in the F_1_ hybrid. A total of 4,697 NAE, 450 over-dominant, and 4,247 dominant genes were detected in the F_1_ hybrid under the WI conditions. Meanwhile, under DS conditions, 1,287 NAE, 40 over-dominant, and 1,247 dominant genes were detected in the F_1_ hybrid (**Figure 3A**). There were 1,143 NAE genes expressed under either WI and DS conditions (**Figure 3B**). Further analysis of these 1,143 NAE genes with the 1,259 DEGs that responded to drought stress in the F_1_ hybrid revealed 403 common genes (**Figure 3B**). These genes were NAE drought stress-specific genes in the F_1_ hybrid. KEGG analysis further revealed that those genes were significantly enriched in the “Biosynthesis of secondary metabolites (ko01110)” and “Glutathione metabolism (ko00480)” pathways (**Figure 3C**), indicating that antioxidant damage-related pathways play an important role in drought response and that there is drought-resistant heterosis in the F_1_ hybrid.

### 3.4 Weighted gene co-expression network analysis

**Figure 4:**
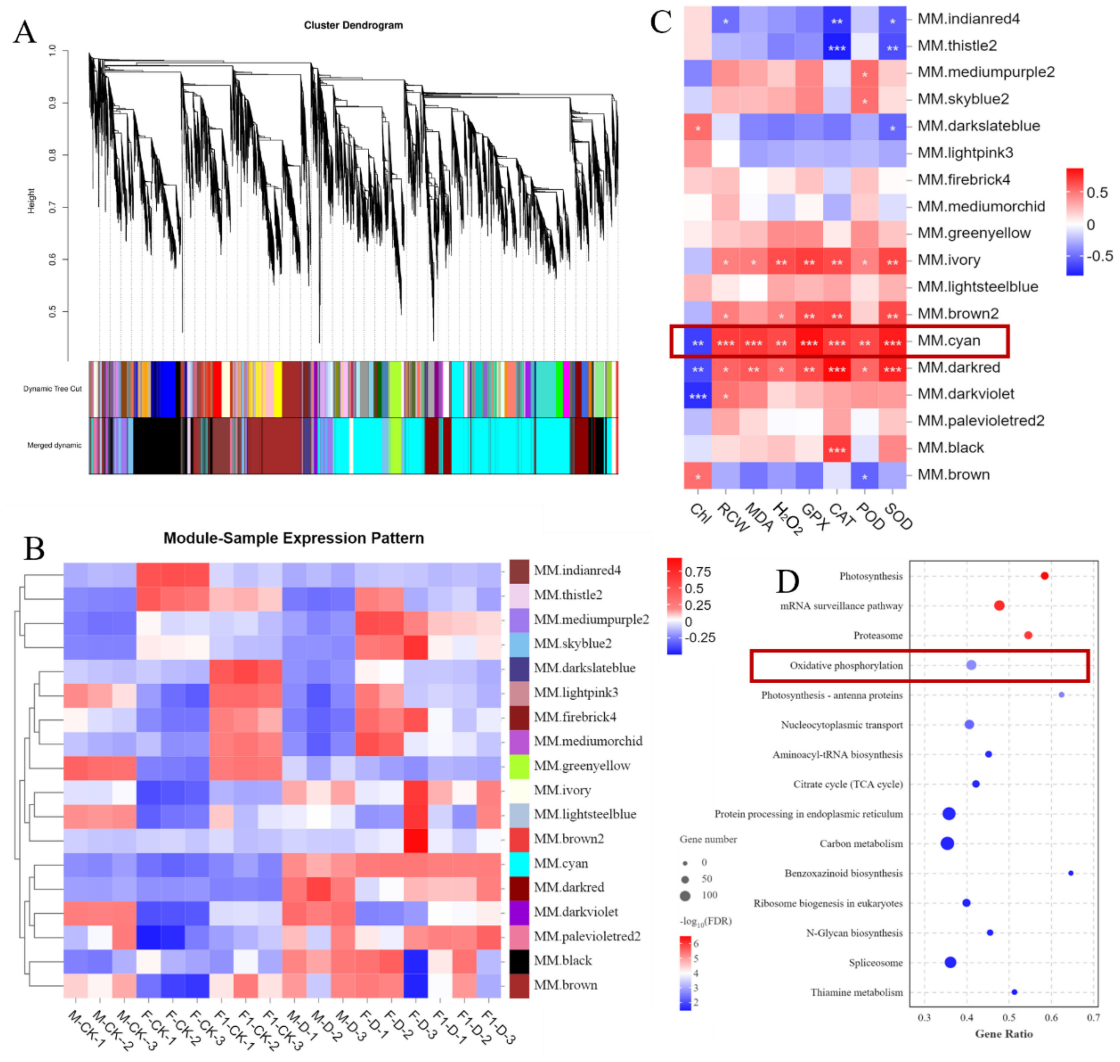
Weighted correlation network analysis of transcriptome data and gene expression associated with response to drought stress. **(A)** Dendrogram of all DEGs clustered based on the measurement of dissimilarity (1-TOM). The color band shows the results obtained from the automatic single-block analysis. **(B)** Heatmap of the correlation between each module eigengene and each sample. **(C)** Heatmap of the correlation between each module eigengene and 8 kinds of physiological and biochemical traits. **(D)** The KEGG pathways (*p* < 0.05) in module cyan. Abbreviations: TOM, topological overlap matrix; ME, module eigengene. student’s *t*-test: *, **, and *** indicate significant levels between each module and traits at 0. 05, 0. 01 and 0. 001 levels respectively.

**Figure 5:**
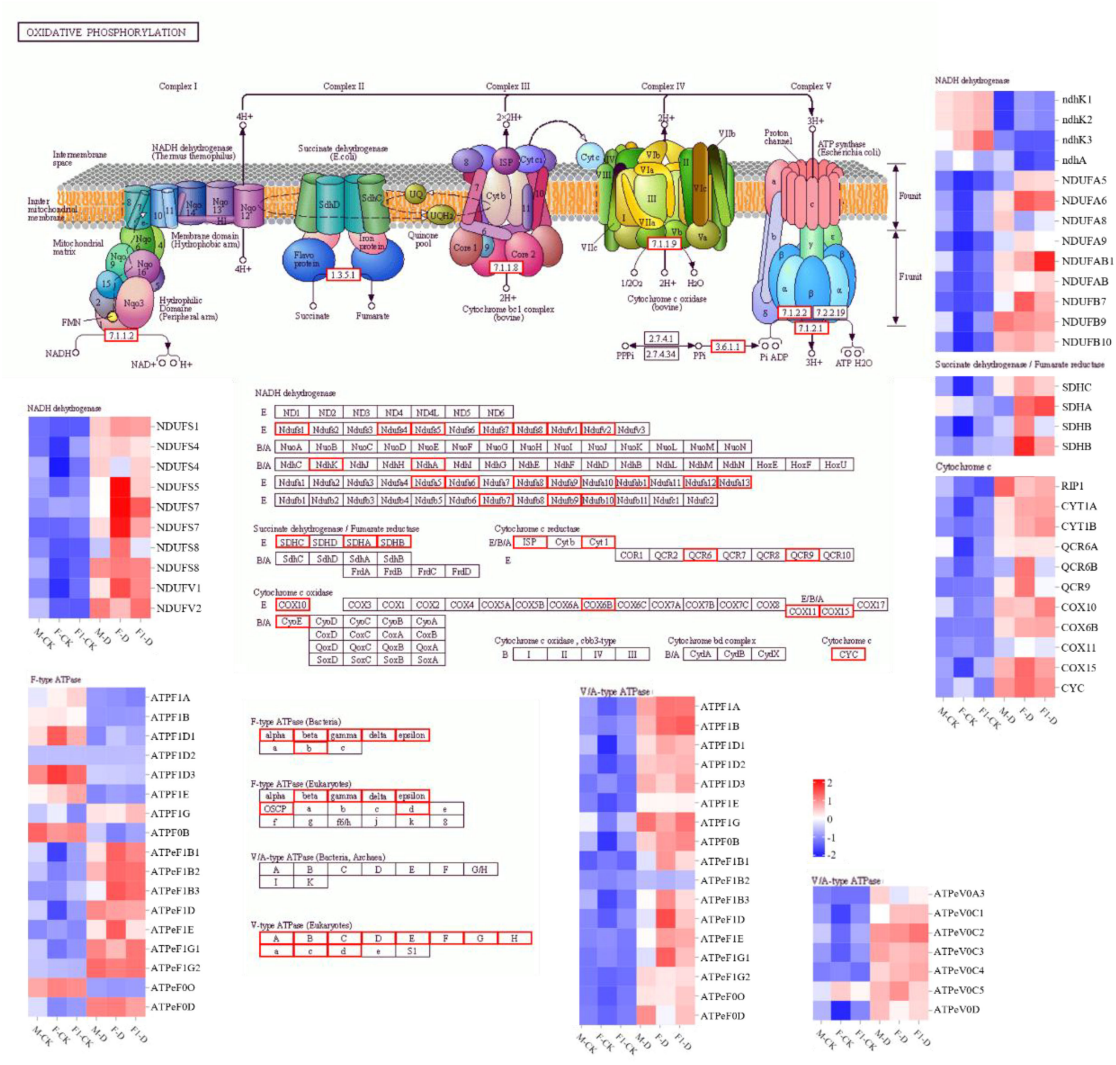
The KEGG map of the oxidative phosphorylation pathway. The analysis of DEGs, comparing drought-treated and control samples among F_1_, F, and M. Boxes in a red frame indicates that the expression of these genes changed before and after drought induction. Heatmap indicates differential expression of genes in each sample as determined by RNA-seq. Treatments are labeled as well-irrigated (WI) and drought-stressed (DS).

To identify genes related to drought-tolerance heterosis, co-expression network analysis was performed on 18 samples using WGCNA, and as a result, 18 modules were obtained (**Figures 4A, B**). Using eight physiological and biochemical indices as trait factors, the correlation between gene modules and traits was analyzed (**Figure 4C**). There was a significant correlation between the MM.black module and CAT indicators. The MM.brown module was significantly correlated with Chl and POD, and the MM.Cyan module was significantly correlated with all indicators (**Figure 4C**). There were 4,073 genes in the Cyan module. KEGG enrichment analysis showed that genes in the Cyan module were mainly enriched in the “Photosynthesis,” “Proteasome,” “Oxidative phosphorylation” and “Photosynthesis - antenna proteins” pathways (**Figure 4D**). Among them, “Proteasome” and “Oxidative phosphorylation”pathways are related to ROS production in plants. One of the main sources of ROS is the mitochondrial respiratory electron transport chain, and mitochondrial complexes I, II, and III are considered to be the main sources of O_2_ production (Waszczak et al., 2018; Nolfi Donegan et al., 2020). According to the KEGG map, genes encoding protease complexes in the oxidative phosphorylation pathway were differentially expressed in response to drought stress induction (**Figure 5**). Among them, the NAD(P)H oxidase *NDHK3* positively regulates ROS generation (Waszczak et al., 2018), and its expression level in the F_1_ hybrid in response to drought stress was lower than that in the parents. The expression of *NDUFS7* and *NDUFAB1*, which are NADH dehydrogenases, reduces the generation of ROS (Hossain and Dietz, 2016), was highly upregulated in the F_1_ plants compared to the parents after drought stress induction, which reflects the ability of F_1_ plants to produce less ROS. This indicates that ROS accumulation plays an important role in the response of maize seedlings to drought stress.

### 3.5 Analysis of candidate genes for drought tolerance heterosis

**Figure 6:**
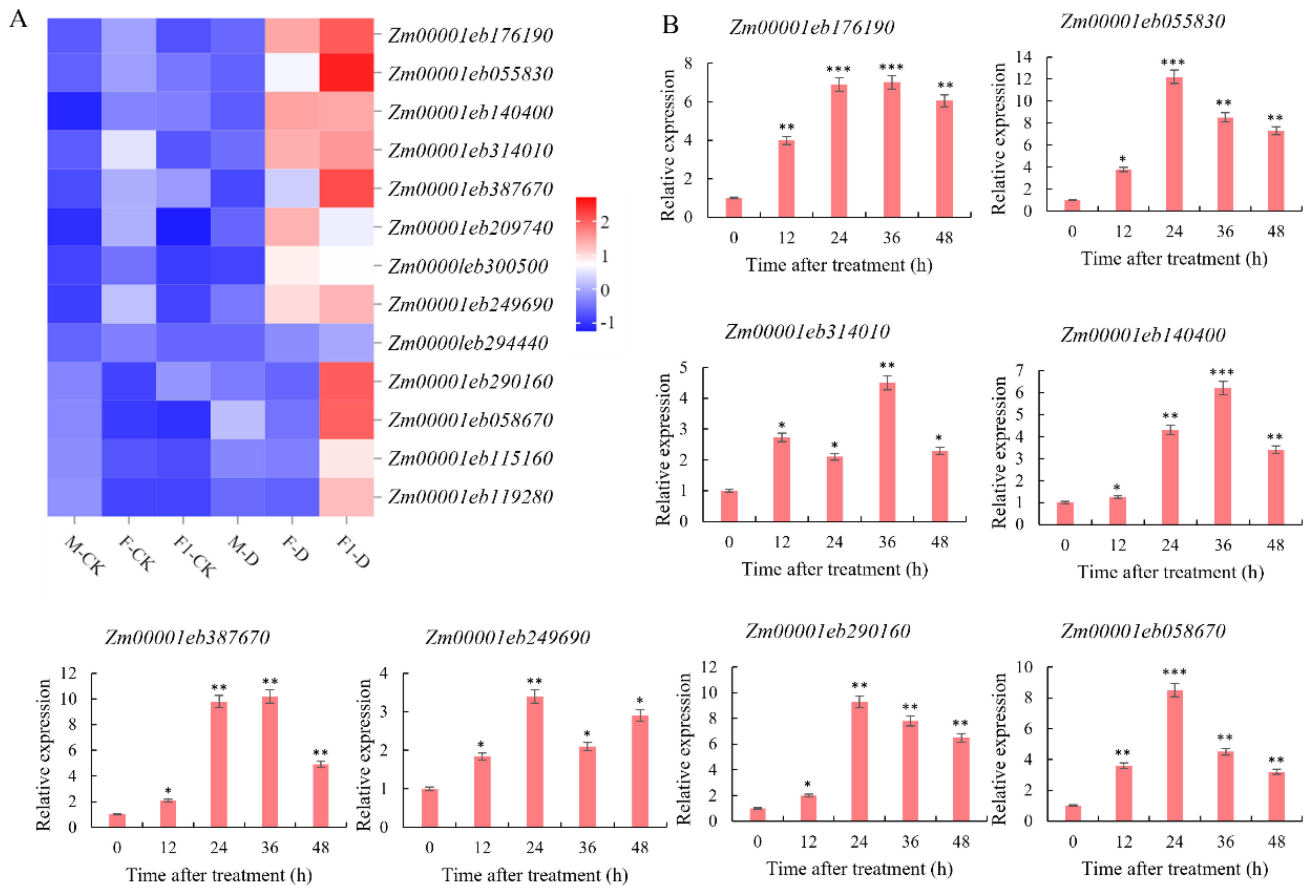
Analysis of candidate gene expression in drought-resistant heterosis. **(A)** Heatmap of the expression of 13 differential genes in each sample as determined by RNA-seq. **(B)** Expression level of 8 genes(*Zm00001eb176190*, *Zm00001eb055830*, *Zm00001eb140400*, *Zm00001eb314010*, *Zm00001eb387670*, *Zm00001eb290160*, *Zm00001eb058670*, *Zm00001eb249690*)under DS conditions. student’s *t*-test: *, **, and *** indicate significant differences between each treatment and 0 h treatment at 0. 05, 0. 01 and 0. 001 levels respectively.

Genes in the Cyan module were compared with the 403 F_1_ genes related to heterosis, and 13 overlapping genes were identified. After drought stress, the expression levels of these 13 genes (*Zm00001eb055830*, *Zm00001eb058670*, *Zm00001eb115160*, *Zm00001eb119280*, *Zm00001eb140400*, *Zm00001eb176190*, *Zm00001eb209740*, *Zm00001eb249690*, *Zm00001eb290160*, *Zm00001eb294440*, *Zm00001eb300500*, *Zm00001eb314010*, *Zm00001eb387670*) in the F_1_ were upregulated. Furthermore, the expression of 7 of those 13 genes (*Zm00001eb055830*, *Zm00001eb140400*, *Zm00001eb176190*, *Zm00001eb209740*, Zm00001eb249690, *Zm00001eb300500*, and *Zm00001eb314010*) were upregulated in the drought-tolerant paternal parent Zheng8713, while the expression levels of the remaining 6 genes showed no significant differential expressions. The expression levels of the 13 genes in the maternal parent showed no significant differences before and after drought stress (**Figure 6A**). These results indicate that these genes played an important role in the response to drought stress and further demonstrate that the paternal parent contributed significantly to the improved drought tolerance of F_1_.

To select candidate genes for drought tolerance heterosis, the expression of eight genes exhibited the highest expression levels in the F_1_ hybrid under WI and DS conditions was analyzed using RT-qPCR (**Figure 6B**). Compared to WI conditions, the expression levels of *Zm00001eb055830* (designated *ZmbHLH137*), *Zm00001eb290160*, and *Zm00001eb058670* continued to increase and peaked at 12, 9.3, and 8.5-fold, respectively, at 24h after drought stress induction. Subsequently, the expression levels were decreased but remained higher than the levels before stress induction. Additionally, *Zm00001eb249690* expression peaked (3.4-fold) at 24 h after drought stress induction, then declined, and increased again at 48 h after drought stress induction. The expression levels of *Zm00001eb176190*, *Zm00001eb140400*, and *Zm00001eb387670* continued to increase after drought stress induction, and all peaked at 7, 6.2, and 10 folds, respectively, at 36 h of drought stress induction, and then declined but remained higher than those before stress induction. The expression of *Zm00001eb314010* was upregulated at 12h of drought stress induction, downregulated at 24 h of stress induction, upregulated again at 36 h, and reached a peak (4.5 folds). In conclusion, *ZmbHLH137* exhibited the highest expression level in drought-stressed seedlings, suggesting that *ZmbHLH137* is an important gene in the regulation of drought-tolerance heterosis in maize.

### 3.6 Functional analysis of the heterosis-related gene *ZmbHLH137*

**Figure 7:**
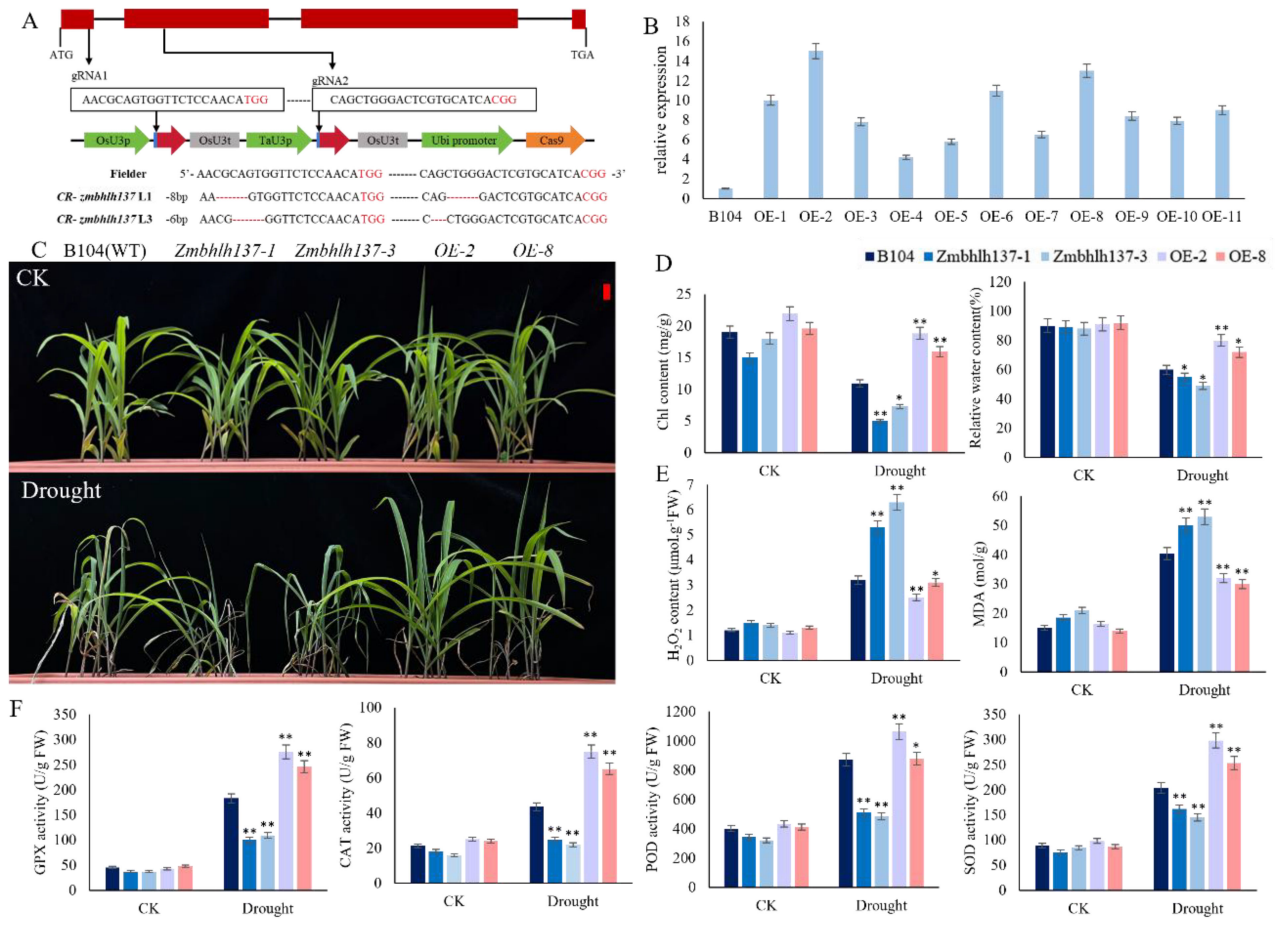
Phenotypic identification and functional characterization of 35::*ZmbHLLH137*-overexpressed (OE-2 and OE-8), B104 (WT), and *zmbhlh137*-knockout mutant (*zmbhlh137–1* and *zmbhlh137-3*) lines under WI and DS conditions. **(A)** Schematic diagram of the mutation site in *zmbhlh137* mutant. **(B)** Expression levels of *ZmbHLLH137* in the OE-2 and OE-8 transgenic lines were detected. **(C)** Seedling phenotypes of the OE, WT, and mutant plants under WI and DS for 7 days. **(D, E)** Relative water content, chlorophyll content, MDA content, and H_2_O_2_ content in the leaf of the OE, WT and mutant plants (n = 3, ± SD). **(F)** Changes of GPX, CAT, POD, and SOD activities in the OE, WT, and mutant plants (n = 3, ± SD). Student’s *t*-test: * and ** represent differences at 0.05 and 0.01 significance levels between the OE, WT, and mutant plants, respectively.

*ZmbHLH137* belongs to the basic helix-loop-helix (bHLH) transcription factor family, which consists of several members and is involved in various plant biological processes including growth, development, and stress responses (Heim et al., 2003). To verify the role of *ZmbHLH137* in drought stress responses, we used CRISPR/Cas9 knockout and transgenic techniques in the maize inbred line B104 (WT) to obtain mutant and overexpression lines. Subsequent drought stress experiments were performed using two knockout mutant lines, i.e., *zmbhlh137–1* and *zmbhlh137-3* (**Figure 7A**), that exhibit translation amino acid loss and frameshift mutations, respectively, and two 35S::*ZmbHLH137*-overexpressing (*ZmbHLH137*-OE), i.e., OE-2 and OE-8 lines (**Figure 7B**). Under WI conditions, all plants grew normally with no significant differences among them. After drought treatment, the 35S::*ZmbHLH137*-overexpressing plants showed the highest growth rate, followed by the WT plants, whereas the mutant plants showed the lowest growth rate (**Figure 7C**). These results indicate that *ZmbHLH137* positively regulates maize drought tolerance at the seedling stage.

After 7 days of drought stress induction, the chlorophyll content and RWC in the 35S::*ZmbHLH137*-overexpressing lines (OE-2 and OE-8) remained normal followed by the WT plants, whereas were significantly decreased in the *zmbhlh137* knockout mutants (**Figure 7D**). Moreover, the MDA and H_2_O_2_ content were significantly increased in all plants in response to drought stress; however, the reduction in the OE-2 and OE-8 lines was significantly lower than that in the WT plants, which were significantly lower than that in the *zmbhlh137* knockout mutants. Drought stress affects the balance of ROS in maize cells and damages the cell membrane; however, the overexpression of *Zmbhlh137* reduces the degree of damage to maize plants. Antioxidant enzymes such as GPX, CAT, POD, and SOD can effectively remove free radicals. The activities of these antioxidant enzymes in maize after drought stress induction were significantly higher than those in plants under non-stressed conditions. Furthermore, 35S::*ZmbHLH137*-overexpressing plants showed significantly higher activities of GPX, CAT, POD, and SOD compared to the WT and *zmbhlh137* mutant plants (**Figures 7E, F**). The results showed that the expression of *ZmbHLH137* significantly enhanced maize drought resistance, decreased ROS accumulation, and increased antioxidant enzyme activity; Knockout of the mutant, on the contrary, confirmed that the gene positively regulates drought resistance by regulating antioxidant pathways.

## 4 Discussion

Maize, an important food crop, is one of the earliest crops to be utilized for heterosis (Chun, 2002). Previous studies have focused on elucidating the phenotypic and molecular mechanisms underlying heterosis associated with growth and development (Song et al., 2016). However, the molecular mechanisms underlying heterosis in maize subjected to abiotic stress remain unclear. This study revealed that the F_1_ hybrid ZhengDan618 was more drought-tolerant compared to its parents and showed heterosis in drought tolerance. Transcriptome sequencing results showed that the drought tolerance phenotype of the F_1_ hybrid is associated with an improvement in antioxidant enzyme activity, with the drought tolerance phenotype of the F_1_ hybrid mostly inherited from the drought-tolerant paternal parent. A non-additive gene abundant in F_1_, *bHLLH137*, positively regulated drought tolerance in maize seedlings by regulating antioxidant enzyme activity.

Previously, a correlation between the drought tolerance of the maize inbred line parents and the drought tolerance of their crossed hybrids was reported (Li, 2011). The fruit difference analysis of *Ginkgo biloba* hybrids showed that seed traits are mainly influenced by the maternal parent, whereas the shape coefficient, fresh weight, and ash content of the hybrid seeds are significantly affected by the parental parent. The total starch content of the seeds was not significantly different from that of the parents. The heredity of the total chlorophyll contents in the hybrid progeny was greatly influenced by the maternal parent, whereas the genetic contribution of the soluble sugar and chlorophyll b contents in the leaves of the offspring were greatly affected by the hybrid paternal parent (Hu, 2023). There was no significant correlation between the drought tolerance of the maize hybrids at the seedling stage and that of their parents. In breeding practices, the drought tolerance of maize hybrids cannot be predicted based on the drought tolerance of their parents alone (Yu et al., 2021). In our study, the phenotype and physiological and biochemical indices were measured. The F_1_ hybrid showed the strongest drought tolerance, and the activities of GPX, CAT, POD, and SOD were strongest in the F_1_ seedlings, followed by the parental parent. Transcriptome analysis showed that the F_1_ hybrid and the parental parent exhibited the lowest number of DEGs, and among the 13 heterosis genes that played a key role in the F_1_ hybrid, seven revealed significantly upregulated expression in the parental parent in response to drought stress, whereas there were no significant DEGs in the maternal parent after drought stress induction. These results suggest a correlation between the drought tolerance phenotype of the F_1_ hybrid and its paternal parent, and that the paternal parent contributes significantly to the improvement of drought tolerance of the F_1_ hybrid.

To survive under stress, plants respond and adapt through complex mechanisms, including developmental, morphological, physiological, and biochemical strategies (Taji et al., 2004; Acosta Motos et al., 2015), involving ion homeostasis, osmolyte biosynthesis, and ROS scavenging systems (Stepien and Klobus, 2005). Under drought conditions, large amounts of ROS are produced in plants, which can cause serious damage to cells. Plants reduce their ROS content by inhibiting ROS production and accelerating ROS clearance (Able et al., 2003). In response to high ROS activity, the antioxidant system in plants, particularly enzymes with antioxidant properties, exerts active oxygen scavenging mechanisms, including GPX, CAT, POD, and SOD activity (Nolfi Donegan et al., 2020). In a previous study on the heterosis of ash (*Fraxinus mandshurica*), it was found that cold-resistant ash hybrids showed significant heterosis in the POD synthetase gene (*FmPOD*) and POD activity (He, 2021). The expression of *DOX1*, which is involved in ROS defense, was upregulated by a factor of 10 and thus plays a potential role in heterosis-associated waterlogging tolerance (Su et al., 2024). Recent studies on the hybrid rice “Liangyoupeijiu” and “Zhegengyou1578” suggested that ROS-related genes are involved in the heterosis of salt tolerance in rice (Huang et al., 2023). The heterosis of the maize hybrid Annong591 was related to ROS genes, and GO analysis revealed that the “peroxisomal matrix (GO:0005782)” and “oxidoreductase activity (GO:0016491)” terms were enriched (Chen, 2021). In the present study, the activities of GPX, CAT, POD, and SOD were evaluated in three maize seedlings under drought-stressed conditions. The activities of these enzymes were highest in the F_1_ hybrid, followed by the paternal parent and maternal parent, with the F_1_ hybrid showing obvious drought tolerance-associated heterosis. Furthermore, the mitochondrial respiratory electron transport chain is an important ROS production pathway in plants, in which mitochondrial complexes I, II, and III are thought to be the main sources of O_2_ (Waszczak et al., 2018). In this study, WGCNA analysis revealed that the genes of all protease complexes in the oxidative phosphorylation pathway were differentially expressed in response to drought stress (**Figure 5**). Among them, the NAD(P)H oxidase *NDHK3* positively regulates ROS generation (Waszczak et al., 2018), and its expression level in the F_1_ hybrid was lower than that in the parents after drought stress induction. The NADH dehydrogenases *NDUFS7* and *NDUFAB1* reduce ROS generation (Hossain and Dietz, 2016). The observed higher expression levels of those genes in the F_1_ hybrid compared to its parents in response to drought stress reflects the ability of F_1_ plants to produce less ROS.

A major goal of agricultural breeding is to produce hybrids that exhibit phenotypes superior to those of their parents. Non-additive genes, including over-dominant and dominant genes, are important for crossbreeding (Zhen et al., 2017). Previous research on the NAE genes in maize revealed that *argos1* (*ZAR1*) and *1‐aminocyclopropane‐1‐carboxylate oxidase2* (*ZmACO2*) contribute to yield-associated heterosis in an over-dominant manner (Wang et al., 2023). A recent study found that the OsWRKY72–OsAAT30/OsGSTU26‐regulatory salinity stress pathway may explain the heterobeltiosis for salinity tolerance exhibited by the hybrid rice line CY1000. Besides, *OsGSTU26* was demonstrated as an important NAE gene in hybrid rice (Liu et al., 2024). In the present study, higher expression levels of 4,697 and 1,287 NAE genes were detected in the F_1_ plants under WI conditions and after drought stress induction, respectively, of which 1,143 genes were highly expressed in the F_1_ plants under both WI and DS conditions. Another comparison with DEGs unique to the F_1_ plants revealed 403 common genes. KEGG analysis showed that they were significantly enriched in the “Biosynthesis of secondary metabolites (ko01110)” and “Glutathione metabolism (ko00480)” pathways. The combined analysis of non-additive genes and WGCNA identified 13 non-additive genes in the F_1_ hybrid in response to drought stress.

Transcription factors (TF) play a crucial role in the signal transduction network, connecting perceived stress signals and subsequent stress responses by altering gene expression (Kong et al., 2020; Shalby et al., 2021). In this study, we targeted the *bHLH137* TF, which is associated with drought heterosis, and found that the expression of *bHLH137* was 12-folds increased in response to drought stress. *bHLH137* is a basic helix-loop-helix (bHLH) protein, its specific domain consists of an alkaline region for DNA binding and an HLH region for dimerization (Zhang et al., 2018). bHLH proteins are found in both animals and plants, with 225, 211, and 308 members of the bHLH protein family found in Arabidopsis, rice, and maize, respectively (Zhang et al., 2018). bHLH TFs bind to the G-box sequence CACGTG and are usually involved in plant growth and development, as well as various abiotic and biological stress responses (Li et al., 2019). Studies on the functions of bHLH TFs in plant responses to drought stress have focused on stomatal development, trichoid development, root hair development, abscisic acid (ABA) sensitivity, and high-temperature-mediated phytochrome interactions (Castilhos et al., 2014). Previous studies have found that the overexpression of *ZmPTF1*, a bHLH family gene expressed in maize, improves the low-phosphorus tolerance of maize. *ZmPTF1* is also a positive regulator of maize root development, ABA synthesis, and signaling pathways, and drought tolerance (Li et al., 2019). Furthermore, *ZmbHLH137* is highly expressed in immature leaves, indicating that it may be involved in plant cell division and elongation (Zhang et al., 2018). Our results showed that *ZmbHLH137* positively regulates the drought tolerance in maize at the seedling stage, and the *ZmbHLH137*-overexpressing plants are more drought tolerant. Analysis of peroxidase activity showed that the activities of the ROS scavenging enzymes GPX, CAT, POD, and SOD were significantly increased in *ZmbHLH137*-overexpressing lines and were significantly lower compared to the WT and *zmbhlh137* knockout mutant lines. The results showed that the overexpression of *ZmbHLH137* activates the antioxidant enzyme system, reduces cell membrane damage, and improves drought tolerance in maize.

In conclusion, the drought tolerance phenotype of the ZhengDan618 F_1_ hybrid is mostly inherited from the drought-tolerant paternal parent, indicating a heterosis-associated drought tolerance phenotype of drought tolerance in maize. Transcriptomic, physiological, and biochemical analyses showed that the drought tolerance heterosis pattern of the ZhengDan618 F_1_ hybrid occurred mainly via the antioxidant pathway. A number of 13 genes related to drought tolerance heterosis were identified, with the *bHLLH137* gene positively regulates drought tolerance in maize seedlings by regulating antioxidant enzyme activity. This study presents the first-ever revelation of the molecular mechanism behind heterosis-associated drought tolerance, which involves the oxidative metabolism pathway, providing novel insights into the regulation of heterosis in maize at the seedling stage via the antioxidant pathway. The identified heterosis genes are valuable genetic assets and can assist in enhancing drought tolerance in maize through strategic approaches. These results hold substantial theoretical and practical importance.

## Data Availability

The original contributions presented in the study are publicly available. This data can be found at the National Center for Biotechnology Information (NCBI) using accession number PRJNA1263706.
