# Supplementary material for: Comprehensive transcriptome analysis provides molecular insights into the heterosis-associated drought tolerance and reveals ZmbHLH137 that promotes drought tolerance in maize seedlings
Source: Front Plant Sci. 2025 May 23;16:1565650. doi: 10.3389/fpls.2025.1565650 (PMC12143235; doi:10.3389/fpls.2025.1565650)
Supplement: Supplementary file 1 [file DataSheet1.doc]

# Supplementary Tables and Figures

**Table S1:** The Primers sequence.

| Primer name | Primer sequence | Purpose |
| --- | --- | --- |
| Zm00001eb055830-F | CGCCCGCGCAAGGCGGCCAA | qRT-PCR |
| Zm00001eb055830-R | CCGCCCAGCTGCGCCGGGTA | qRT-PCR |
| Zm00001eb058670-F | GTGCTCTTCGACACCTCGCT | qRT-PCR |
| Zm00001eb058670-R | CTCAAGGCGCTCCTCTTCTA | qRT-PCR |
| Zm00001eb115160-F | GTGGCGAACCTGCTGGGCCT | qRT-PCR |
| Zm00001eb115160-R | CGTTCGACGTCAAGTACCTG | qRT-PCR |
| Zm00001eb119280-F | AACGGCGTCGGAGGGCTTCA | qRT-PCR |
| Zm00001eb119280-R | TTCAAACCCAGCAGCAACAT | qRT-PCR |
| Zm00001eb140400-F | TCCAGGTCCTGCTGCGGGTG | qRT-PCR |
| Zm00001eb140400-R | CGGCTGCCCACGCCGACTAC | qRT-PCR |
| Zm00001eb176190-F | TGGAGATTGTTTTCCGAGCT | qRT-PCR |
| Zm00001eb176190-R | TGAGGCGAACTGGGCGATGA | qRT-PCR |
| Zm00001eb209740-F | GGTTCGCGTCCAAGATCGAG | qRT-PCR |
| Zm00001eb209740-R | TTCGCTAGCTCGGCCAACAC | qRT-PCR |
| Zm00001eb249690-F | CGAAGATCCTGCTTACACTG | qRT-PCR |
| Zm00001eb249690-R | CGATCCCGACAAAGCGTTCA | qRT-PCR |
| Zm00001eb290160-F | ACGACGACGCCACGGGCCGC | qRT-PCR |
| Zm00001eb290160-R | TTCCTCGCCGTCAGGGCCTT | qRT-PCR |
| Zm00001eb290160-F | CACGGCCACGCGCACGGTGT | qRT-PCR |
| Zm00001eb290160-R | TGGTGCACTCGCTGATCATC | qRT-PCR |
| Zm00001eb294440-F | ACACCACGCTGGCCCGTGGG | qRT-PCR |
| Zm00001eb294440-R | AAGTTCAATGAGGCGAAATA | qRT-PCR |
| Zm00001eb300500-F | CACCAGGTTCGCCACGCCGG | qRT-PCR |
| Zm00001eb300500-R | ACGCGTCGGGCGCGTCGGGC | qRT-PCR |
| Zm00001eb314010-F | TTCGCCGCTACGTGCAGCCT | qRT-PCR |
| Zm00001eb314010-R | ACCACCATGAACTTGCTGTC | qRT-PCR |
| 18S-F | CCTGCGGCTTAATTGACTC | qRT-PCR |
| 18S-R | GTTAGCAGGCTGAGGTCTGG | qRT-PCR |
| ZmbHLH137-F1 | AATAATGGTCTCAGGCGCACCGCCCGCGCAAGGCGGCCAA | knockout |
| ZmbHLH137-F2 | GCACCGCCCGCGCAAGGCGGCCAAGTTTTAGAGCTAGAAATAGC | knockout |
| ZmbHLH137-R1 | AACTACCCGGCGCAGCTGGGCGGGCTTCTTGGTGCC | knockout |
| ZmbHLH137-R2 | ATTATTGGTCTCTAAACTACCCGGCGCAGCTGGGCGG | knockout |
| ZmbHLH137‑pFGC5941‑F | TTGGCGCGCCATGGCAACGCAGTGGTTCTC | overexpression |
| ZmbHLH137‑pFGC5941‑R | CGGGATCCCTGAAGGATGATTTGAACGCTT | overexpression |

**Table S2:** The statistics of mRNA sequenced libraries.

| **Sample** | **Raw reads** | **Clean reads (%)** | **AF_Q20(%)** | **AF_Q30(%)** |
| --- | --- | --- | --- | --- |
| M-CK-1 | 45829202 | 45538848 (99.37%) | 6560517130(96.34%) | 6157735043(90.43%) |
| M-CK-2 | 49770180 | 49413336(99.28%) | 7097771994 (96.04%) | 6648719108(89.96%) |
| M-CK-3 | 44674060 | 44383896(99.35%) | 6398418357 (96.47%) | 6017162693(90.72%) |
| F-CK-1 | 40998882 | 40719628(99.32%) | 5878104496 (96.45%) | 5522974595(90.62%) |
| F-CK-2 | 52128706 | 51786370(99.34%) | 7452297797 (96.22%) | 6993068310(90.29%) |
| F-CK-3 | 45077726 | 44772756(99.32%) | 6442840326 (96.18%) | 6043535371(90.22%) |
| F1-CK-1 | 39619696 | 39383414(99.40%) | 5661111565 (96.13%) | 5307440071(90.12%) |
| F1-CK-2 | 43122662 | 42858936(99.39%) | 6167834859 (96.14%) | 5781013722(90.11%) |
| F1-CK-3 | 47181750 | 46879068(99.36%) | 6711530757 (95.75%) | 6264717611(89.38%) |
| M-D-1 | 46029520 | 45723766(99.34%) | 6598345108 (96.46%) | 6211704919 (90.80%) |
| M-D-2 | 40294688 | 40013362(99.30%) | 5767032246 (96.36%) | 5424510580 (90.63%) |
| M-D-3 | 44910704 | 44569640(99.24%) | 6404981360 (96.07%) | 6001508657 (90.02%) |
| F-D-1 | 47790404 | 47408614(99.20%) | 6802354708 (95.87%) | 6363992428 (89.69%) |
| F-D-2 | 42727256 | 42377790(99.18%) | 6084680391 (95.97%) | 5701154001 (89.92%) |
| F-D-3 | 58751454 | 58628762(99.79%) | 8303183766 (96.78%) | 7857043328 (91.58%) |
| F1-D-1 | 48453876 | 48331182(99.75%) | 6950686321 (96.17%) | 6525339360 (90.29%) |
| F1-D-2 | 44454242 | 44318434(99.69%) | 6384825487 (96.26%) | 6000267061 (90.46%) |
| F1-D-3 | 42084516 | 41923010 99.62%) | 6020178069 (96.04%) | 5645382267 (90.06%) |


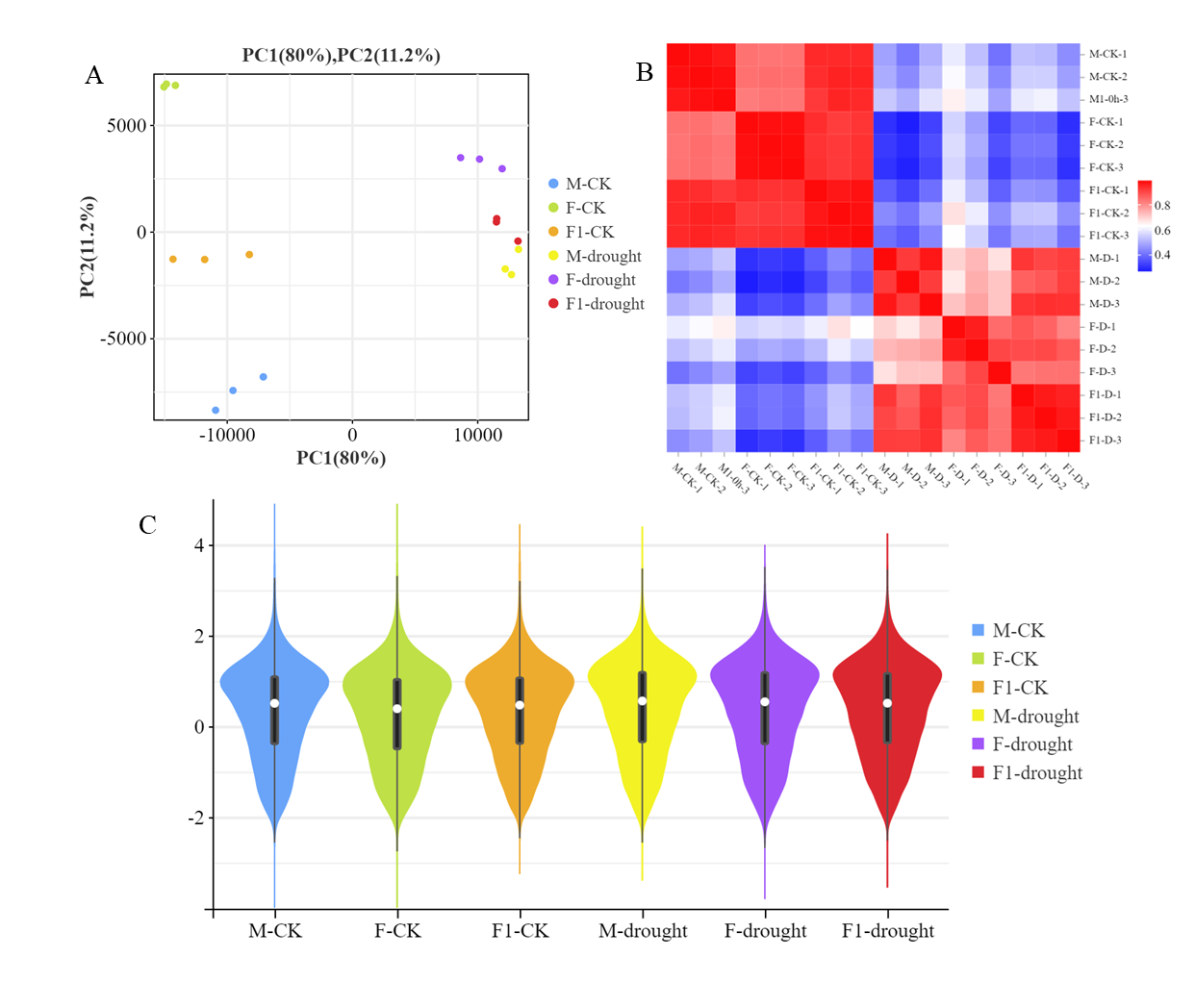


**Fig. S1. Quality analysis of transcriptome data.** (**A**)The principal components analysis of RNA-seq data under drought-stressed conditions (**B**) Pearson correlation analysis of three biological replicates. (**C**) Box plot of the FPKM distribution of the 18 samples.
